# Motivations and satisfaction of sports tourists during the FIFA world cup in Qatar 2022

**DOI:** 10.1016/j.heliyon.2024.e26682

**Published:** 2024-02-19

**Authors:** Mauricio Carvache-Franco, Tahani Hassan, Miguel Orden-Mejía, Orly Carvache-Franco, Wilmer Carvache-Franco

**Affiliations:** a Universidad Bolivariana del Ecuador, Campus Durán Km 5.5 vía Durán Yaguachi, Durán, 092405, Ecuador; b Universidad Espíritu Santo-Ecuador, Km. 2.5 Vía a Samborondón, Samborondón, Ecuador; c Brunel University London, Brunel Business School, Kingston Lane, Uxbridge, Middlesex UB8 3PH, London, United Kingdom; d Universitat Rovira i Virgili, Facultat de Turisme i Geografia, Carrer Joanot Martorell, 15, Vila-seca, Spain; e Universidad Católica de Santiago de Guayaquil, Facultad de Ingeniería, Km 1.5 Av. Carlos Julio Arosemena, Guayaquil, Ecuador; f Escuela Superior Politécnica del Litoral, ESPOL. Facultad de Ciencias Sociales y Humanísticas, Campus Gustavo Galindo Km 30.5 Vía Perimetral, P.O. Box 09-01-5863, Guayaquil, Ecuador

**Keywords:** Soccer world cups, Sports tourism, Motivations, Satisfaction

## Abstract

This study aimed to identify the motivations of sports tourists to attend Soccer World Cups and to determine the motivational dimensions that predict their satisfaction. The research took place in Doha, the capital of Qatar, during the FIFA World Cup in 2022, where 503 valid questionnaires were collected. Exploratory factor analysis and linear regression with the Enter method were used for data analysis. The results show four motivational dimensions: Sports Passion, Escape & Relaxation, Patriotism & Attachment, and Recreation. Additionally, a positive and significant relationship was found between the Escape & Relaxation factor and overall satisfaction. In contrast, a negative correlation was identified between the Patriotism & Attachment dimension and satisfaction. These findings will contribute to the academic literature related to the Soccer World Cups as part of sports tourism and will also assist sporting event organizers in developing marketing and management plans to benefit this tourist demand.

## 1 Introduction

The sports tourism sector has experienced significant growth during the new millennium, primarily attributed to the dissemination efforts by the UNWTO [1] at the Barcelona conference in 2021 and the increasing importance of sports tourism. Consequently, researchers have taken a keen interest in studying this type of tourism comprehensively, aiming to understand its various aspects and provide solutions to its challenges [2].

The rising interest in investing in sports tourism has led to countries competing for the opportunity to host such events, recognizing their potential to boost the economy, enhance the destination's image, build its brand, and create significant social and cultural impacts [[3], [4], [5], [6], [7], [8], [9]]. Therefore, studying sports tourism thoroughly and considering all angles is crucial to ensure better organization and management of future sporting events. Over the years, the tourism sector has experienced significant development, encompassing various activities, including sports. There is a growing interest among countries to host sporting events due to their impact on elevating the destination's image and generating substantial revenue [10]. Sports tourism can be defined as the driving factors or reasons that lead spectators to attend a sporting event in another country [11].

The primary determinant of tourist trips is the motivation of the tourists themselves. In tourism, motivations represent clusters of psychological factors or needs that drive individuals to fulfill their desire to visit a destination or engage in leisure-related activities [12]. Understanding these motivations is vital in the tourism industry as they play a crucial role in enhancing the destination's image, helping in the planning and implementing effective strategies, and increasing tourist satisfaction with the destination [13]. Different motivations or factors influence visiting particular destinations to attend sporting events. Hence, studying and understanding these travel motivations is crucial as they can lead to an improved appreciation of the host country's culture, tourist attractions, and overall destination offerings. Visiting a destination and the likelihood of returning are closely tied to visitor satisfaction. Understanding these two variables can offer valuable information on visitors' spending patterns and facilitate the organization of future sporting events by optimizing facilities and improving program plans [14].

The 2022 FIFA World Cup marked a historic event as it was hosted by Qatar, making it the first Arab and Middle Eastern country to hold this prestigious sporting tournament. Qatar is located in the Arabian Gulf and gained independence from the United Kingdom in 1971 [15,16]. This country shares borders with Bahrain, Saudi Arabia, and the United Arab Emirates. The total area of Qatar is 11,571 square kilometers, and its capital is Doha. As of 2020, its population reached 2881 million [17]. Its climate is characterized by hot and humid summers, contrasted with cooler winters [18].

Islam serves as the official religion of Qatar, with the presence of various religious groups, including Christians and Hindu workers who speak English as their second language. However, Arabic remains the primary language spoken in Qatar [17]. As for Qatar's economy, it relies on oil and gas exports, but recently, it has taken steps to diversify its revenue sources. These efforts include encouraging foreign investment and liberalizing the economy [17].

Researching sports tourism events, particularly world soccer championships like the 2022 World Cup in Qatar, holds significant importance for several reasons. Firstly, the field of sports tourism is still struggling to advance theoretically [19], and there is a need for more comprehensive studies in this area. Secondly, this is the first mega-sport event that occurs in the area of the Middle East, which has different characteristics than other areas of the world in terms of its geography, population, traditions, economy, religion, and political set-up, which may affect the organization of the event, its activities, and tourists views of the hosting country and the event itself. As a result, this may affect the visitors' views, motivations, and satisfaction with their visit. Finally, according to the researchers' knowledge, this is the first study to investigate the 2022 World Cup in Qatar concerning the motivations and satisfaction of tourists. The available studies were only focused on stadium construction, opportunities and challenges for hosting the 2022 FIFA World Cup, motivations for volunteering for the FIFA 2022 World Cup in Qatar, and the cultural impact of Qatar hosting the World Cup as the first Arab World Cup [[20], [21], [22], [23], [24], [25]].

Despite soccer's immense popularity and impact on society, the economy, politics, and culture, academic attention towards soccer events and their relevance to the tourism sector remains limited. The existing literature indicates a scarcity of studies focusing on soccer events and their significance for sports tourism [26,27]. In light of this knowledge gap, studying soccer-based tourism becomes crucial. Therefore, the present study on the FIFA World Cup 2022 in Qatar aims to achieve two main objectives: (1) Identify the motivations of sports tourists to attend a world soccer championship, and (2) determine the motivational dimensions that predict their satisfaction with these events.

This study contributes to the academic literature on sports tourism, specifically focusing on visitors to the World Cup and other mega-soccer events worldwide. This research achieves two primary objectives: firstly, by identifying the motivational factors that influence visitors' decisions to travel to Qatar, and secondly, by expanding the understanding of how these motivational factors impact the overall satisfaction with sports tourists. In practical terms, the findings of this study can serve as a management guide for sporting event managers and organizers. By gaining insights into the motivational factors that drive sports tourists to attend these events, managers can develop plans targeted to the tourist demand. Thus, understating can improve various services, including accommodation, transportation, and security, to ensure tourist satisfaction.

## 2 Literature

### 2.1 Motivations in the context of soccer tourism

Unveiling the motivation of tourists to attend soccer events plays a crucial role in developing the travel industry. This type of tourism substantially impacts the host destination through the sports program, the activities surrounding the sporting event, and the overall tourist environment created by the organizers. Sporting events can shape the destination's image and market the location based on its guests' attractions, culture, and social aspects [28]. Within the realm of sports tourism, various offerings cater to the interests of travelers. These include sporting events, complexes, sports cruises, sports attractions, sports adventures, and sports tours. Collectively, these categories encompass the utilization of sports for tourism activities, providing travelers with diverse experiences centered around their sporting interests [29].

Soccer, one of the most widely followed sports globally, attracts many spectators to matches held anywhere in the world. Thus, it demands meticulous planning and organization to maximize income generation, with tourism among the sectors with a keen interest in this industry. Soccer tourism involves various stakeholders, including spectators, leagues, federations, clubs, players, coaches, cities, regions, countries, local authorities, transport, shops, museums, accommodations, food and drink establishments, sponsors, and media entities. It is characterized by spectators visiting a destination to witness soccer matches, attend soccer team training sessions, and enjoy watching their favorite soccer teams and players in action [27]. While soccer tourism holds significant importance, it can come with some negative impacts on stakeholders, such as overcrowding, noise, pollution, and traffic congestion, which requires organizers to deploy police, security personnel, and volunteer workers to ensure smooth organization and maintain security during the events [27,30].

Studying the motivations and factors that drive tourists to attend soccer sporting events is of utmost importance as it enables a comprehensive understanding of their decision-making process. This understanding, in turn, empowers organizers and marketers to strategize and plan current and future events to align with the desires and needs of tourists based on their specific motives [11].

From a broader perspective, motivations represent a significant state of mind that compels individuals or groups to travel [31]. In sports tourism, motivations are crucial in tourists' intention to attend a sporting event [32]. Tourists may have different reasons driving their decision to travel to participate in sporting events. For example, Mishra et al. [32] identified two main motivations for attending active sporting events in India and Poland: personal motives, including travel exploration, self-improvement, stress relief, physical strength, and social motives, encompassing social bonds and social recognition. Similarly, Khong et al. [33], studying the Monsoon Cup and the Malaysian Grand Prix and Motorcycling events, identified the following reasons for attending these events: knowledge about culture and people, sociability, relaxation, entertainment, and prestige.

The literature on travel motivations to attend soccer matches has revealed different factors that may vary from one event to another, influencing the decisions of sports tourists. For instance, in a study focusing on small football events, Elahi, Moradi & Saffari [34] identified the following motivations for tourists attending Esteghlal and Persepolis matches in the 2016–2017 Iranian Premier Football League: connection to the team, escape, esthetics and excitement. Similarly, a study on the motivation of tourists to attend England soccer team matches in the Europa League 2019/20 found 11 reasons for travel: witnessing the team's achievement, the drama of watching a fantastic game, entertainment, escape and relaxation, fear of missing out, gourmet -trying local food and drink-, kinship, learning about the place and its culture, seeing the opposing team, socialization and the stimulation of engaging in something exciting [35]. A recent study found that motivations to attend football matches vary among individuals regarding their occupation. For instance, Namethee et al. [36] found that students' motivations to attend football matches are namely enjoyment, friendship, relaxation, “belonging and self-esteem, and “fandom."

Studies on the motivations for attending major sporting events, such as the World Cup, have identified several factors. For example, Andersson, Bengtsson, & Svensson [10] studied the motivations to attend the UEFA European Football Championship 2016 in France, the FIFA World Cup 2018 in Russia, and the FIFA World Cup 2022 in Qatar. They found three key reasons: interest in soccer, a good feeling when leaving, and fun things to do with family and friends. Assaker & Reis [37] found that tourists visiting Fortaleza were motivated by entertainment, enjoyment, supporting their country's team, the importance of the game's outcome, and seeing the best soccer players in the world. Before the World Cup 2022 in Qatar, Yahia, I. B. and Al-Emadi, M [38]. distinguished between “pull factors,” including cultural attractions, security, road safety, facilities, visual attractions (scenery), and uniqueness of the destination; and, “push factors,” which involved motives for traveling, such as escape and novelty, as well as and the motives for attending the event, related to support and passion. Finally, in a study on the Sepak Takraw event in Malaysia (volleyball, soccer, martial arts, and gymnastics event), Zarei and Ramkissoon [39] identified eight factors for attending, including economic, group affiliation, self-esteem, stress, entertainment, escape, family, and aesthetics.

Based on the previous discussion, researchers have no consensus on visitor motivations to attend sporting events, particularly in the context of soccer matches. Diverse factors can drive tourists, including the size of the event, its significance, its program, the host country, and the available facilities catering to their wants and needs. The previous points on the motives behind soccer tourism have prompted our first research question.

RQ1 What are the motivations of sports tourists to attend a world soccer championship?

### 2.2 Satisfaction in the context of soccer tourism

Satisfaction is widely recognized as a pivotal concept in tourism and is one of the most extensively researched aspects [40]. Satisfaction can be generally defined as the extent to which an experience is perceived to produce favorable sensations [41]. Tourism satisfaction encompasses tourists' overall feelings toward particular domains in their vacation experience [42]. In sports tourism, previous studies have defined satisfaction during a sporting event as a pleasurable and satisfactory response to the sports competition and complementary services provided during a match [37].

A thorough examination of the sports tourism literature has revealed a gap in studies addressing the influence of motivations on satisfaction in the context of soccer tourism. This demonstrates the insufficient investigation of the impact of motivation on satisfaction in soccer tourism, which shows the need for further research to understand this influence comprehensively. Thus, our second research question emerges.

RQ2 What motivational dimensions predict the satisfaction of sports tourists during world soccer championships?

## 3 FIFA world cup 2022 in Qatar

Qatar, a small nation with a land area of less than 12,000 km^2^ and a population of 2.7 million, is located on the northeast coast of the Arabian Peninsula [43], sharing borders with Bahrain and Saudi Arabia. Almost 90% of its population consists of migrants [43]. The oil and gas industry is the Qatari economy's cornerstone, with significant reserves recently discovered [43]. The FIFA World Cup 2022 in Qatar marked a historic milestone as the first hosted by an Arab country and the second in Asia, standing as the most extraordinary 2022 event. Statistical data indicates that the World Cup attracted over 1.4 million fans from across the globe [44], including the final match, where Argentina defeated France and emerged as the winner [45]. Visitors enjoyed the tournament, with more than 530,000 people attending the daily entertainment events between matches, including the FIFA Fan Festival and Corniche Activation [44].

Qatar pursues several objectives in hosting prestigious sporting events such as the 2022 FIFA World Cup and the upcoming 2023 Football Asian Cup, as outlined by Reiche [46]: (1) to foster better international relations; (2) promote national health; (3) establish Qatar as a world sports center; (4) enhance their international reputation; (5) promote national cohesion; and (6) update its infrastructure. Qatar built four new stadiums for the FIFA World Cup and renovated four existing ones. Notably, the main stadium has an impressive capacity of 86,000 people. These preparations extended beyond the sporting venues, with infrastructure improvements in the host cities. Projects included implementing new rail and metro systems connecting the three host cities [43]. Additionally, Doha airport's annual passenger capacity was expanded from 30 to 50 million, and Qatar constructed 240 new hotels, focusing on categories 4 and 5 [43]. The modernization of the Port of Doah with hotels, restaurants, and tourist entertainment attractions further contributed to the enhancement of the area [43].

Organizing the World Cup in Qatar posed unique challenges, primarily due to the high temperatures that prevailed during June and July when prior World Cup tournaments were held [20,43,47]. As a result, the competition was rescheduled to November and December for the first time in the tournament's history. In addition, Qatar installed air conditioning in the stadiums to ensure a comfortable environment for foreign visitors unaccustomed to high temperatures [48,49].

Qatar's cultural and Arab religious traditions presented additional social obstacles [47]. Balancing these traditions with the expectations of the millions of viewers, some of whom may have different customs, was essential to avoid potential cultural clashes. An example is the prohibition of alcohol consumption in Qatar, which could have conflicted with post-results celebrations typical in many other countries.

To promote cultural exchange, awareness, and non-discrimination, Qatar embarked on a multifaceted approach to foster respect, knowledge, and tolerance for traditions [47], combining its 2030 National Vision, the World Cup, and the development of its capital city, Doha [50]. During the World Cup, tourists could engage in many different activities. For those interested in art and culture, the Museum of Islamic Art looks at artifacts dating back to the time of Islam and unique books and restaurants. The National Museum of Qatar opened in 2019 and has information on Qatar's history, with more than 15,000 artifacts from Sheikh Faisal's collection. Finally, the Sheikh Faisal Bin Qassim Al Thani Museum presents the pre-oil habits of the region [51]. Shoppers can explore Souq Waqif, Doha's largest market, open from 10 a.m. to 10 p.m., offering traditional Qatari clothing, food, art, jewelry, animals, home decor items, and souvenirs [51]. Msheireb Downtown Doha, a planned neighborhood in central Qatar, features 100 buildings linked by 10,000 underground parking spaces, with many high-end shops, restaurants, and a unique ambiance. Another attraction is The Pearl, a luxurious planned island representing Doha's second commercial district, with upscale shopping, dining, scenic coastlines, and recreational activities, such as aquatic taxis, movies, and outdoor restaurants, making it a delightful destination for visitors to explore and enjoy [51].

## 4 Method

The present study had ethical approval from the ESPOL Polytechnic University of Ecuador Code: FCSH-14- 2021. The informed consent was obtained in writing from all participants, and acceptance was requested from each respondent at the beginning of the questionnaire.

### 4.1 Participants

The participants were foreign tourists visiting Qatar to attend the 2022 World Cup soccer matches. They were people over 18 years of age. Additionally, the cumulative attendance at stadiums in Qatar was 3.4 million spectators, an increase compared to the World Cup in Russia, which reached 3 million fans.

### 4.2 Questionnaire design

The present study employed a structured questionnaire consisting of three sections: sociodemographic aspects, motivations for attending the FIFA World Cup 2022 in Qatar, and satisfaction. The first part included thirteen closed-ended questions focusing on the sociodemographic characteristics of the attendees obtained from Lee et al.'s study [52]. In the second section, the participants' motivations were analyzed using a set of 16 items taken from Zarei and Ramkissoon [39]. These items were rated on a 5-point Likert-type scale, ranging from 1, “very little,” to 5, “a lot.” The third part measured satisfaction with the sporting event and consisted of three satisfaction-related questions adapted from the study by Kim & Park [53].

Initially, the survey was translated by three multilingual researchers from the tourism and hospitality industries, aiming to cater to the target audience's specific needs. The survey, first drafted in English, was translated into English and Arabic versions. These versions were subsequently uploaded onto the Google Forms Program. Following this, a pilot test was carried out with 25 participants, and the findings from this trial guided some improvements to enhance the quality of the survey responses. These participants were chosen from among the tourists who will begin arriving in the city of Doha, the capital of Qatar, before the start of the 2022 World Cup.

These adjustments included examining the validity of the survey to ensure it accurately measured the intended factors and assessing the time required for completion. Finally, the final survey was made available on Google Forms and tailored to the online participation of those attending the World Cup 2022 in Qatar.

### 4.3 Data collection

A group of surveyors were on site to ask World Cup attendees to fill out the survey on WhatsApp. Sample collection occurred between November 20 and December 18, 2022, in Doha, Qatar, a city known for its significant Muslim population and many expatriate communities. Respondents were invited to participate in the survey while engaging in recreational activities at the World Cup, such as visits to museums, restaurants, and tourist attractions. Respondents received all necessary information about the importance of the survey. Then, they were asked for their WhatsApp number, and the link to the survey was shared. Informed consent was included in the survey. Six hundred surveys were sent, and 503 valid questionnaires were received, using a 5% margin of error, 95% level of confidence, and 50% variance. It was not necessary to reweight the data collected from the sample.

A significant majority of the respondents were men (63.2%). The age distribution fell within the 30–50 years range, primarily representing Millennials and Generation X groups, the most prevalent attendees of the World Cup. The vast majority of respondents possessed higher education qualifications. In terms of marital status, both single and married individuals were nearly equal in numbers. Over a third of the surveyed individuals reported being employed in a private organization. Additionally, the majority of the respondents in Qatar were of European origin. (See Table 1),

**Table 1:** Sociodemographic aspects.

Characteristics	Distribution	Frequency	%
Gender	Male	318	63.2
	Female	185	36.8
Age	<20	86	17.1
	21–30	156	31
	31–40	117	23.3
	41–50	96	19.1
	51–60	39	7.8
	>61	9	1.8
Education Level	Primary	14	2.8
	Secondary	150	29.8
	University	332	66
	Postgraduate/Master/PhD	7	1.4
Marital Status	Single	212	42.1
	Married	211	41.9
	Others	80	15.9
Occupation	Student	85	16.9
	Businessman	16	3.2
	Researcher/Scientist	4	0.8
	Private employee	197	39.2
	Public employee	96	19.1
	Pensioner	32	6.4
	Unemployed	53	10.5
	Other	20	4
Nationality	Saudi	37	7.4
	Kuwaiti	1	0.2
	Foreign	465	92.4
Origin	Asian	56	11.1
	European	171	34
	North American	62	12.3
	South American	58	11.5
	Rest of the world	156	31

According to a CNN News report [54], the State of Qatar's Ministry of Public Health established specific standards to guarantee a COVID-19-free event. Additionally, it's imperative to constantly wear a mask when using public transit, especially taxis, and to be aware that fewer individuals will be permitted inside the service at any time to allow for social separation. Before you enter the stadiums and boarding public transportation, your temperature will be taken. Also, you could be required to present your Hayya Card. To enter the nation for the 2022 FIFA World Cup, you must have a Hayya Card [54]. As a result, the survey's collector took health precautions to ensure that it complied with the guidelines established by Qatar's Ministry of Health regarding mask wear and social distancing.

### 4.4 Analysis of data

Data interpretation and item comprehension were facilitated through factor analysis, with a restricted number of factors employed. The Principal Components Method was used as an extraction technique, and factor loadings were ordered using the Varimax rotation technique. The number of appropriate factors was determined using the Kaiser model.

The evaluation of the proposed model included applying Bartlett's test of sphericity and the KMO index (Kaiser-Meyer-Olkin). Linear regression was used with the Enter method to discern the specific motivational factors that predict overall satisfaction in the context of the Soccer World Cup. Gpower, Jasp, and SPSS software organized categorized, and tabulated the data.

## 5 Results

### 5.1 Motivations in soccer world cups

The factorability of the data was analyzed to validate the questionnaire. Before conducting the factor analysis, the Kaiser-Meyer-Olkin (KMO) test was applied to measure the sampling adequacy of the data. This test helps determine whether the observed variables are appropriate for extracting underlying factors. The KMO value obtained for this study was 0.754, surpassing the recommended threshold of 0.6, which indicates that the data were suitable for factorization. Similarly, Bartlett's Test of Sphericity (=5175.876; df = 120; p < 0.001) determined that the samples did not have equal variances, allowing for a factor analysis with these values.

A principal components analysis with orthogonal rotation (Varimax) was conducted on the 20 variables that measured the reasons for attending the Soccer World Cup. After the rotation, a four-factor model was extracted, explaining 67% of the variance. The criteria for factor loadings equal to or greater than 0.4 were applied in this model. Four variables, namely, “To escape from the routine,” “To be with friends,” “To be with my family,” and “To enjoy hotels and restaurants,” were eliminated as they did not meet the specified criteria. This resulted in 16 variables, showing substantial factor loadings that were grouped into four constructs: Sports Passion, Escape & Relaxation, Patriotism & Attachment, and Recreation. The psychometric properties of these four constructs demonstrated a robust structure that can be considered intrinsic/extrinsic factors within sports motivations for attending a World Cup.

In addition, we examined Cronbach's Alpha (α) coefficients and the values for Kurtosis and Skewness for each construct. The results were as follows: Sports Passion (Skewness = −0.800; Kurtosis = 0.874; α = 0.762), Escape & Relaxation (Skewness = −0.114; Kurtosis = −1.120; α = 0.891), Patriotism & Attachment (Skewness = −1.660; Kurtosis = 2.114; α = 0.7 12), and Recreation (Skewness = −1.590; Kurtosis = 2.767; α = 0.753). According to Byrne [55], values within the range of +3 and −3 suggest normally distributed data. Additionally, for a construct to exhibit good reliability and internal consistency, Cronbach's Alpha values must surpass the 0.7 threshold. In our study, the overall value of Cronbach's Alpha was 0.765, indicating satisfactory reliability. Therefore, the factors demonstrate both univariate normality and reliability.

### 5.2 Common method bias (CMB)

We used "Harman's single factor test” to assess the presence of CMB, employing a maximum likelihood method as outlined in previous research [56,57]. This approach, using SPSS, allowed us to analyze potential CMB. According to Harman's criteria, if the variance explained by a single factor exceeds 50%, it suggests the existence of CMB in the dataset. Our results showed no risk of CMB, as the percentage of variance attributed to a single construct was 23.37, which falls below the 50% threshold. Furthermore, we employed a full collinearity approach to examine CMB. The obtained values were as follows: Sports Passion = 1,510, Escape & Relaxation = 1,010, Patriotism & Attachment = 2198 and Recreation = 1869.

Factor 1, called Sports Passion, explains 23.4% of the total variance, with a unit vector 3.74. Their love for soccer drives this motivation as tourists travel to the World Cup to attend the games, enjoy the artistry of the players on the field, join the lively crowd of fans, and share the experience with friends, all with the primary purpose of supporting their team through songs and chants. These results indicate that Sports Passion in soccer is multifaceted, encompassing a deep love for the sport, appreciation for its artistic elements, enjoyment of live match experiences, camaraderie with fellow fans, and the social aspect of sharing these experiences with friends.

The results indicate that Factor 2, labeled “Escape & Relaxation,” accounts for 21.4% of the total variance, suggesting that it is a significant factor in explaining the motivations of individuals attending the World Cup. The unit vector of 3.42 indicates the strength and direction of this factor.

The motivations driving this factor are primarily focused on seeking an escape from daily problems and adding excitement to one's life through the experience of attending the World Cup. Individuals view the event as a way to temporarily disconnect from their worries and enjoy themselves, ultimately seeking feelings of excitement and happiness.

Factor 3, Patriotism & Attachment, explains 14.4% of the total variance, with a unit vector 2308. This motivation involves a deep sense of national belonging and attachment. Tourists express patriotism by wearing the national colors, singing the national anthem, displaying country flags and banners, and demonstrating unwavering support for the team, regardless of match outcomes. These results indicate that Patriotism and attachment are essential factors motivating individuals to attend the World Cup. It reflects a deep-seated sense of national pride and identity and a desire to celebrate and support one's country on the global soccer stage.

Factor 4, Recreation, explains 7.8% of the total variance, with a unit vector 1.24. Beyond the sport, this motivation revolves around the recreational aspects of the World Cup. Tourists are drawn to the fun-filled activities before, during, and after each soccer match, making it an enjoyable event. Recreation is an essential factor motivating individuals to attend the World Cup. It reflects a desire for entertainment and fun and an appreciation for the overall experience and atmosphere surrounding the tournament beyond just the sport itself. (See Table 2, Table 3).

**Table 2:** Component loads.

Factor	Items	Loading				Uniqueness
Sports Passion	Because soccer is important to me	0.864				0.283
	To enjoy soccer as an art	0.842				0.277
	To attend soccer matches	0.819				0.353
	To be with fans	0.632				0.535
	To join crowds	0.532				0.507
	For fun with friends	0.503				0.621
Escape & Relaxation	To be excited		0.893			0.194
	To feel good		0.892			0.168
	To forget about problems		0.864			0.231
	To add excitement to my life		0.816			0.329
	For the culture			0.845		0.130
Patriotism & Attachment	To get to know Qatar			0.842		0.132
	For patriotism			0.832		0.249
	To be there for the victories and defeats of my team			0.803		0.259
Recreation	To enjoy the activities and events				0.677	0.502
	For recreation				0.644	0.514

Bartlett's Test: $x^{2}$ = 5175.876; *df* = 120; p < 0.001. Chi-squared Test: Value = 1520.083; *df* = 62; p < 00.001. Kaiser– Meyer–Olkin Test = 0.754.

**Table 3:** Component characteristics.

	Unrotated solution			Rotated solution		
	Eigenvalue	Proportion var.	Cumulative	SumSq. Loadings	Proportion var.	Cumulative
Component 1	3.740	0.234	0.234	3.308	0.207	0.207
Component 2	3.426	0.214	0.448	3.057	0.191	0.398
Component 3	2.308	0.144	0.592	2.897	0.181	0.579
Component 4	1.243	0.078	0.670	1.456	0.091	0.670

These results answer our first research question, RQ1. What are the motivations of sports tourists to attend a world soccer championship?

### 5.3 Motivation and satisfaction with soccer world cups

To predict the impact of motivational factors (independent variable IV) on overall satisfaction at the World Cup (dependent variable DV), a linear regression analysis was conducted using the Enter method. The resulting coefficients from this analysis represent the magnitude and direction of the relationship between IV and DV. In the study, the F ratio, collinearity, and data independence values were acceptable in the model (See Table 4).

**Table 4:** Relationship between motivation variables and satisfaction variables.

Model	B	Standard error	*β*	*t*	p	Tolerance	VIF
Motivations							
Sports Passion	−0.012	0.016	−0.031	−0.72	0.467	1.000	1.000
Escape & Relaxation	0.064	0.016	0.169	3.915	<0.001	1.000	1.000
Patriotism & Attachment	−0.072	0.016	−0.191	−4.42	<0.001	1.000	1.000
Recreation	0.014	0.016	0.036	0.830	0.407	1.000	1.000

Note: Durbin-Watson = 2.130; *F*-statistic = 9.029; $R^{2}$ = 0.046; Adj. $R^{2}$ = 0.060; 1- *β* = 0.97; $f^{2}$ = 0.072.

The Escape & Relaxation factors positively and significantly correlated with overall satisfaction. A World Cup is an exciting event filled with energy and enjoyment. The matches, the vibrant stadium atmosphere, the pre and post-match festivities, and the camaraderie of fellow fans can create a gratifying experience full of emotion and enthusiasm, contributing to satisfaction.

In the same way, traveling and immersing oneself in soccer can serve as a means of escape from daily worries, allowing individuals to disconnect from daily problems and responsibilities. This can produce temporary relief and a sense of relaxation, contributing to satisfaction.

In addition, participating as a fan in soccer matches, often alongside people from different cultures, can offer an enriching experience, which will help to add variety to the daily routine. For some tourists, this exposure to the host country's culture, traditions, and customs becomes a source of satisfaction, further enhancing their experience.

The findings also revealed a negative correlation between Patriotism & Attachment and satisfaction. These results could be associated with expectations of the soccer team's performance. Even the desire to be present at all stages, both victories and defeats, could generate tensions and disappointments. Additionally, this behavior could be due to personal expectations regarding fun, excitement, and overall event enjoyment.

On the other hand, Sports Passion and Recreation yield insignificant results. Strict restrictions by the organizing country could have influenced tourist satisfaction. If restrictions prevent tourists from actively participating in certain football-related events or activities, those with a great passion for the sport could feel limited and less satisfied. The restrictions could affect the sporting experience and the opportunity for tourists to explore, recreate, and enjoy local culture and other tourist activities and attractions, influencing their overall satisfaction. These findings answer our second research question: RQ2: What motivational dimensions predict the satisfaction of sports tourists during world soccer championships?

## 6 Conclusions

Sports tourism involves tourists visiting a destination to attend sporting events organized and sponsored by that destination. Among the numerous sporting events held worldwide, the Soccer World Cup stands out as a mega sporting event for which countries compete to host. The status of soccer fuels this competition, which is considered the most popular game globally. Millions of people worldwide spend hours watching soccer matches on television, purchasing tickets to attend the games, and staying updated on soccer news and its star players. Therefore, hosting the Soccer World Cups can attract millions of tourists to the host country to witness the matches. This presents an excellent opportunity for visitors to immerse themselves in the host country's culture, history, cuisine, and tourist attractions, effectively positioning the nation as a desirable tourist and sports destination. Ultimately, this boosts the host country's revenue and enhances its international reputation.

The present research aimed to study the motivations driving tourists to attend a soccer World Cup and to identify the factors influencing their overall satisfaction, using Qatar 2022 as a case study to contribute to the academic literature on sports tourism. The findings unveiled four distinct motivational dimensions within the context of Soccer World Cups and sports tourism: Sports Passion, Escape & Relaxation, Patriotism & Attachment, and Recreation. Notably, the study revealed a positive and significant correlation between the Escape & Relaxation dimension and overall satisfaction, highlighting how soccer-related experiences temporarily escape daily problems and responsibilities. Conversely, a negative association was observed between the Patriotism & Attachment dimension and overall satisfaction.

### 6.1 Theoretical implications

The primary objective of this study was to identify the motivations of sports tourists to attend a world soccer championship. In response to RQ1, the results revealed four motivational dimensions: Sports passion, Escape & Relaxation, Patriotism & Attachment, and Recreation. Similar findings were observed in previous studies concerning the first dimension, Sports Passion. Andersson, Bengtsson, & Svensson [10] identified this factor as “an interest in soccer,” while Assaker & Reis [37] found it as “watching the best soccer players in the world."

The second factor found was Escape & Relaxation. This motivation was also recognized in previous research. Elahi, Moradi & Saffari [34], Reimers, Chao & Speechley [58], and Zarei and Ramkissoon [39] all characterized it only as “escape.” Bason [35] found it under the same name, referring to it as “escape and relaxation.” Finally, Yahia and Al-Emadi [38] identified it as “escape and novelty.” Meanwhile, Namethee et al. [36] found it only as “Relaxation."

The third factor, Patriotism & Attachment, was not consistently found in the same way in previous findings. Yahia and Al-Emadi [38] called it “support and passion.” Other authors highlighted aspects of connection or identification with the team, such as Elahi, Moradi & Saffari [34] and Reimers, Chao & Speechley [58]. Additionally, Bason [35] found this dimension to be “seeing the team's achievement."

The fourth factor found was Recreation. This motivation aligns with previous studies where it was identified as an “entertaining” dimension by Zarei and Ramkissoon [39]. Similarly, Andersson, Bengtsson, & Svensson [10] recognized it as “fun things to do with family and friends,” and Brown, Assaker & Reis [37] as “excitement."

This study makes a significant contribution to the academic literature by identifying a set of motivational dimensions in sports tourism specifically applied to world soccer championships. By integrating four distinct motivations, Sports Passion, Escape & Relaxation, Patriotism & Attachment, and Recreation, this research goes beyond previous findings that often identified similar motivations but treated them in isolation. In this study, the interplay of these motivations highlights the multifaceted nature of reasons for attending these sporting events, enriching our understanding of the complex factors that drive sports tourists. Importantly, this research identifies motivations not previously found in the literature, such as Sports Passion, and Patriotism & Attachment. These dimensions highlight the profound influence of sporting enthusiasm and emotional attachment to the team and country, significantly impacting the tourism experience in the context of world soccer championships.

As a second objective, this research aimed to determine the motivations that predict the satisfaction of sports tourists in a world soccer event. In this sense, addressing RQ2, the results revealed a positive and significant relationship between the Escape & Relaxation factor and overall satisfaction. This relationship suggests that for many sports tourists, attending a world soccer event offers a unique way to escape from the daily routine and disconnect from everyday problems. On the other hand, the results showed a negative relationship between the Patriotism & Attachment factor and satisfaction. This suggests that fans who have strong emotional attachments to their soccer team may experience a decline in satisfaction if their team faces losses or disappointments during the tournament. Notably, the literature has been relatively silent on the specific impacts of individual motivational dimensions on overall satisfaction in the context of World Soccer Cups. While previous studies, such as Assaker and Reis [37], have highlighted the positive impact of motivations on satisfaction, this research goes a step further by disentangling the distinct effects of each motivational factor on satisfaction. In this respect, having identified that the Escape & Relaxation dimension positively affects satisfaction and that the Patriotism & Attachment dimension impacts it negatively, this study offers a significant contribution to the academic literature on sports tourism, providing a nuanced understanding of how different motivational dimensions shape the overall satisfaction of sports tourists attending world soccer championships.

As theoretical implications, this study contributes to the literature by presenting four motivational dimensions, providing a comprehensive framework that includes the sporting, social, and cultural aspects inherent in trips to World Cups. Additionally, two novel dimensions, Sports Passion and Patriotism & Attachment, have emerged, expanding the understanding of motivations in this context.

### 6.2 Practical implications

As practical implications, this study recommends that to enhance the Sports Passion motivational dimension, World Cup host destinations ensure the necessary facilities for attending sporting events, such as designated areas in bars of each country with minimal waiting times. Additionally, having support staff in the stadiums to assist attendees is essential. To improve the Escape & Relaxation motivational dimension, implementing tourist packages with exciting activities linked to the sports event is advisable. Also, promoting the event internationally through social media channels, highlighting the sports aspects and the opportunity for various leisure activities, could further enhance the overall experience.

Concerning the Patriotism & Attachment motivational dimension, it is recommended that fans at the destination, regardless of their team's performance, feel enthusiastic and have an enjoyable leisure experience. World soccer event organizers could conduct campaigns encouraging fans to explore the city, savor its local cuisine, and partake in tourist activities. Diversifying Soccer World Cup tour packages with various visits and leisure activities is increasingly important. Regarding the Recreation motivational dimension, host destinations should consider incorporating artistic and cultural events and festivals, tour operators could offer full-day excursions to sites near the sporting event, restaurants and entertainment venues could introduce artistic performances, and city tours should be adapted to the visitors' needs. Furthermore, a blend of parties, contests, and recreational activities can also be implemented in the host cities of these sporting events.

The Escape & Relaxation motivation positively influences satisfaction with the World Cup. Therefore, this dimension could be improved by organizing leisure events and stadium attendance. Additionally, offering tourism packages with various recreational activities in the destination and promoting the Soccer World Cup as an event where attendees can enjoy the matches and explore and appreciate the host location is advisable. Given the negative relationship between Patriotism & Attachment and satisfaction, it is recommended to conduct campaigns promoting recreation and tourism in the host cities. These campaigns should encourage people to relax during their stay and make the most of leisure sites, restaurants, hotels, and entertainment venues, which can help mitigate the impact of the team's match results on overall satisfaction. All the implications derived from the research will provide valuable management guidelines for companies and institutions involved in organizing soccer world cups.

### 6.3 Limitations and future line of research

It is essential to acknowledge certain limitations in this study. The margin of error and the utilization of a convenience sampling method may have impacted the sample's representativeness. Another limiting factor was the timing of the data collection during the actual sporting event, which could have influenced the respondent's perceptions. Despite these limitations, the findings contribute significantly to the sports tourism literature and offer actionable insights for industry stakeholders, including World Cup organizers and tourism service providers. Finally, further research could delve into segmenting the demand for Soccer World Cups based on motivational factors. If we investigate the demand segments, we could develop products according to each group of tourists found.

## Data availability statement

The data is shared along with the manuscript.

## CRediT authorship contribution statement

**Mauricio Carvache-Franco:** Writing – review & editing, Writing – original draft, Visualization, Validation, Methodology, Investigation, Formal analysis, Data curation, Conceptualization. **Tahani Hassan:** Writing – review & editing, Writing – original draft, Visualization, Validation, Methodology, Investigation, Formal analysis, Data curation, Conceptualization. **Miguel Orden-Mejía:** Writing – review & editing, Writing – original draft, Visualization, Validation, Methodology, Investigation, Formal analysis, Data curation, Conceptualization. **Orly Carvache-Franco:** Writing – review & editing, Writing – original draft, Visualization, Validation, Software, Methodology, Investigation, Formal analysis, Data curation, Conceptualization. **Wilmer Carvache-Franco:** Writing – review & editing, Writing – original draft, Visualization, Validation, Methodology, Investigation, Formal analysis, Data curation, Conceptualization.

## Declaration of competing interest

The authors declare that they have no known competing financial interests or personal relationships that could have appeared to influence the work reported in this paper.
